# Early ablation leads to better outcome in patients < 55 years with persistent atrial fibrillation

**DOI:** 10.1038/s41598-024-76098-2

**Published:** 2024-10-25

**Authors:** Nico Erhard, Fabian Bahlke, Bruno Neuner, Miruna Popa, Hannah Krafft, Alexander Tunsch-Martinez, Jan Syväri, Madeleine Tydecks, Edison Abdiu, Marta Telishevska, Sarah Lengauer, Gabriele Hessling, Isabel Deisenhofer, Florian Englert

**Affiliations:** 1 Department of Electrophysiology, German Heart Center Munich, Technical University of Munich, Lazarettstraße 36, 80636 Munich, Germany; 2 Department of Anesthesiology and Intensive Care Medicine, Charité-Universitätsmedizin Berlin, Berlin, Germany

**Keywords:** Persistent atrial fibrillation, Diagnosis-to-ablation time, Catheter ablation, Young patients, Cardiology, Interventional cardiology

## Abstract

The question of optimal timing for catheter ablation of atrial fibrillation (AF) to achieve best outcomes remains a crucial clinical issue. As AF occurs less frequently in younger patients, data regarding Diagnosis-to-Ablation Time (DAT) is especially limited in patients under the age of 55 years with persistent AF. We therefore analyzed the temporal relationship between initial AF presentation and timing of catheter ablation in this cohort. We conducted a retrospective single-centre study of patients ≤ 55 years with persistent AF who underwent first-time catheter ablation at our center. The cohort was divided into patients that underwent catheter ablation after diagnosis of persistent AF within a DAT of ≤ 12 months and patients with a DAT of > 12 months. A total of 101 patients (median age 51 years; female *n* = 19 (18.8%)) with persistent AF were included. Ablation was performed within 12 months (“early DAT”) in 51 patients and > 12 months (“late DAT”) in 50 patients. Pulmonary vein isolation was performed using high-power short-duration (HPSD) radiofrequency ablation. Median DAT was 5 months (1–12 months) in the early ablation group and 36 months (13–240 months) in the late ablation group. The median follow-up was 11.3 months (0.03–37.1 months). The rate of any atrial arrhythmia recurrence after a 30-day blanking period was significantly lower in the early DAT group (13/51 patients; 25.5%) as compared to the late DAT group (26/50 patients; 52.0%) (log rank test; *p* = 0.003). Catheter ablation performed > 12 months after the initial AF diagnosis was an independent predictor for the occurrence of any atrial arrythmia (OR: 2.58; (95%-CI: 1.32–5.07). Early first-time catheter ablation (DAT ≤ 12 months) in patients ≤ 55 years with persistent AF is associated with a significantly lower rate of arrhythmia recurrence.

## Introduction

Although atrial fibrillation (AF) is becoming increasingly prevalent also in a younger patient population^1^, persistent AF is still quite rare in individuals under the age of 55 years and frequently linked to recognizable causes such as structural heart abnormalities, hyperthyroidism, or excessive alcohol consumption^1,2^.

Early rhythm-control therapy is linked to a reduced likelihood of cardiovascular events in patients with early-stage AF^3^. Catheter ablation has emerged as a standard strategy for treating AF, but the optimal timing for the intervention remains an important question with regard to improving ablation outcomes^4,5^. While numerous large studies have reported ablation outcomes in older patients, data focusing on younger patients with persistent AF is limited^2,6^.

We therefore conducted a retrospective analysis in a younger patient cohort (< 55 years) that presented with persistent AF and underwent first time ablation therapy. We sought to analyze the temporal relationship between initial AF presentation and the timing of catheter ablation in respect of long—term outcome.

## Methods

### Study design

This retrospective single center study included a cohort of 101 patients ≤ 55 years (at time of ablation) who were diagnosed with persistent AF and underwent first-time radiofrequency catheter ablation (RFCA) at our center between January 2017 and December 2020. Persistent AF was defined according to current guidelines^4^. Patients with previous left atrial catheter ablations, other atrial arrythmias, with congenital heart defect (excluding PFO and minor ASD) were excluded from the study. Patients were divided in those who underwent ablation within 12 months after AF diagnosis or > 12 months after AF diagnosis. Primary outcome was ablation success, defined as the absence of AF recurrence. Procedure-related complications occurring within 30 days of ablation were also assessed.

Ethical approval was granted from the Institutional Review Board (ethical committee of the technical university of Munich, (approval #2020 − 348_1-S-NP). Due to the retrospective nature of the study, (ethical committee of the technical university of Munich) waived the need of obtaining informed consent.

All methods were performed in accordance with the relevant guidelines and regulations as well as in accordance with the Declaration of Helsinki.

## Data collection

Demographic information, clinical characteristics, procedural details, and follow-up outcomes were extracted from the center´s computerized database. Ablation success was defined as the absence of AF recurrence without the need for antiarrhythmic drugs, assessed on 3-, 6-, and 12-month follow-up visits using 7-day Holter ECGs (after a 30-day blanking period post-ablation). In the initial 30 days post-ablation, complications were evaluated based on in-patient surveillance and subsequent follow-up consultations at 1 month or during any unplanned appointments.

## Ablation procedure

All patients were under uninterrupted oral anticoagulation at least 4 weeks prior the procedure and 3 months afterwards. Antiarrhythmic medications (AAD) were halted more than five half-lives before the procedure and weren’t resumed post-procedure. Before undergoing ablation, and within a 48-hour timeframe, the absence of left atrial thrombus was confirmed through contrast-enhanced cardiac computed tomography (CT). If there were reasons preventing the use of contrast agents or if a CT scan wasn’t available, transesophageal echocardiography was utilized instead. During the procedure, CT segmentations, which displayed the left atrial anatomy and the relative position of the esophagus to the left atrial posterior wall, were available. The ablation procedure was executed under monitored conscious sedation, employing a regimen of midazolam, propofol, and fentanyl. The femoral access was secured utilizing an anatomically guided approach.

Electroanatomical high-density mapping was executed employing either the EnSiteTM Velocity, EnSiteTM Precision (Abbott) or the CARTO^®^ 3 system (Biosense Webster, Irvine, CA). For wide antral circumferential pulmonary vein isolation (PVI), point-by-point lesions were created either with the 4 mm irrigated-tip FlexAbilityTM SE catheter coupled with the Ampere^®^ RF generator (Abbott) or the 3.5 mm irrigated-tip Thermocool Smarttouch^®^ SF catheter paired with the SmartAblate RF generator (Biosense Webster). The ablation catheter was not positioned within the steerable sheath. Ablation was performed a using high power short duration (HPSD) protocol as previously published by our group^7–9^.

Energy setting were set to 70 W/7 s (anterior wall) or 70 W/5 s (posterior wall) with the FlexAbilityTM SE catheter, while parameters of 60 W/10 s (anterior wall) or 60 W/7 s (posterior wall) were employed with the Thermocool Smarttouch SF catheter. The precise power and temporal configurations were grounded on in silico and ex vivo examinations, as delineated in previous studies by Bourier et al.^8^. Additional linear lesions or substrate modification were left to the discretion of the operating physician.

Transthoracic echocardiography was employed to assess pericardial effusion immediately after the procedure, the subsequent morning, and in instances of hemodynamic instability. A post-procedural echocardiographic detection of a new effusion with a dimension ≥ 10 mm was deemed significant pericardial effusion. All patients received a proton pump inhibitor (pantoprazole 40 mg twice daily) for 4 weeks.

Following the ablation oral anticoagulation was continued for at least 3 months. Betablockers were continued if tolerated by the patient. Other AAD were discontinued in all patients except for an arrythmia recurrence within the first 2 days following the ablation procedure. In case of an early arrhythmia recurrence during the blanking period, electrical cardioversion was performed.

### Statistical analysis

Continuous variables are expressed as mean (standard deviation) and differences between two independent groups evaluated by Student’s *t*-tests. Binary and categorical variables are presented as absolute (n) and relative (%) frequencies. Differences between two independent groups were tested by chi-squared test or by Fisher’s exact test. Time to first AF/AT recurrence was plotted using the Kaplan–Meier method and differences in survival time between two independent groups compared by the logrank test. The proportional hazard assumption was tested as a prerequisite of applying the logrank test. Univariant cox regression analysis was performed to identify potential variables associated with arrythmia recurrences. A two-sided p-value of < 0.05 was considered statistically significant. Data were analyzed using SPSS software version 28.0 (IBM Corp. Released 2021. IBM SPSS Statistics for Windows, Version 28.0. Armonk, NY: IBM Corp)) and R (R foundation for statistical computing).

## Results

### Patient population

A total of 101 patients with persistent AF underwent ablation with a DAT ≤ 12 months (*n* = 51) or > 12 months (*n* = 50). Median DAT was 5 months^1–11^ in the early ablation group and 36 months (13–240) in the late ablation group.

Baseline characteristics were well-balanced between groups and are presented in Table 1. Mean age (50 y (38–55) vs. 51.5 y (26–55) *p* = 0.76), female gender [5 (9.8%) vs. 14 (28.0%) *p* = 0.019], mean BMI (31.6 kg/m^2^ (5.8) vs. 29.2 kg/m^2^ (5.0) *p* = 0.062), mean LVEF (58% (23–65) vs. 59% (26–65) *p* = 0.53), Left atrium size (25.7 cm^2^ (5.8) vs. 26.8 cm^2^ (6.9) *p* = 0.85) were similar between the groups. The proportion of patients suffering from hypertension, diabetes, patients with amiodarone intake and non-smokers was also comparable. Patients with coronary heart disease were more often in the early ablation group (5 (9.8%) vs. 1 (2.0%) *p* = 0.21). Congenital heart defect (persistent foramen ovale, atrial septum defect) was rare (3 (5.9%) vs.6 (12.0%) *p* = 0.32) and equally distributed between the groups.

**Table 1:** Baseline patient characteristics.

	All patients *n* = 101	Ablation within 12 months *n* = 51	Ablation after 12 months *n* = 50	*p*-value
DAT in months, median (range)	12 (1–240)	5.0 (1–12)	36 (13–240)	**< 0.001**
Female gender, n (%)	19 (18.8%)	5 (9.8%)	14 (28.0%)	**0.019**
Age in years, median (range)	51 (26–55)	50 (38–55)	51.5 (26–55)	0.76
Body mass index in kg/m2, mean (SD)	30.4 (5.5)	31.6 (5.8)	29.2 (5.0)	0.062
Hypertension, n (%)	53 (52.5%)	30 (58.8%)	23 (46.0%)	0.20
Diabetes mellitus, n (%)	7 (6.9%)	5 (9.8%)	2 (4.0%)	0.44^#^
Coronary heart disease, n (%)	6 (5.9%)	5 (9.8%)	1 (2.0%)	0.21^#^
Congenital heart defect, n (%)	9 (8.9%)	3 (5.9%)	6 (12.0%)	0.32^#^
Tobacco use, n (%) (*n* = 63)				0.065^&^
None	45 (71.4%)	23 (71.9%)	22 (71.0%)	
Current	11 (17.5%)	8 (25.0%)	3 (9.7%)	
Ex-smoker over 10 PY	7 (11.1%)	1 (3.1%)	6 (19.4%)	
Beta blockers, n (%)	90 (89.1%)	46 (90.2%)	44 (88.0%)	0.72
Amiodarone, n (%)	3 (3.0%)	1 (2.0%)	2 (4.0%)	0.62^#^
Ejection fraction in %, median (range)	58 (23–65)	58 (23–65)	59 (26–65)	0.53
Left atrium, diameter in mm, mean (SD)	43.7 (6.8)	43.7 (7.1)	43.7 (6.7)	0.40
Left atrium, size in cm2, mean (SD)	26.2 (6.3)	25.7 (5.8)	26.8 (6.9)	0.85

# = Fisher’s exact test; & = Fisher-Freeman-Halton exact test.

## Procedural characteristics

Data concerning the ablation procedure is summarized in Table 2. Mean energy in watts was similar (55.8 (7.6) vs.54.9 (6.6) *p* = 0.44), also RF time in minutes, median (22.0 (9.9–43.3) vs.21.8 (7.8–63.5) *p* = 0.58), mean Temperature in °C (30.2 (4.4) vs. 28.9 (4.0) *p* = 0.30) and fluoroscopy time (8.5 min (1.3–24.1) vs. 7.2 min (1.4–32.1) *p* = 0.34) were similar. X-ray dose was higher in the early ablation group (287 Gy (33–10.373) vs. 165 Gy (12–1985) *p* = 0.016). Additional substrate modification including ablation lines were also comparable between the groups.

**Table 2:** Procedural data.

	All patients *n* = 101	Ablation within 12 months *n* = 51	Ablation after 12 months *n* = 50	*p*-value
Energy in Watts, mean (SD)	55.4 (7.1)	55.8 (7.6)	54.9 (6.6)	0.44
RF time in minutes, median (Range)	21.9 (7.8–63.5)	22.0 (9.9–43.3)	21.8 (7.8–63.5)	0.58
Temperature in °C, mean (SD)	29.6 (4.3)	30.2 (4.4)	28.9 (4.0)	0.30
X-ray dose in gray, median (range)	246 (12–10.373)	287 (33–10.373)	165 (12–1985)	**0.016**ara>
Fluoroscopy in min, median (range)	8.1 (1.3–32.1)	8.5 (1.3–24.1)	7.2 (1.4–32.1)	0.34
Atrial tachycardia intra-procedural, n (%)				0.87^&^
None	90 (89.1%)	46 (90.2%)	44 (88.0%)	
atyp. A.-flutt.	9 (8.9%)	4 (7.8%)	5 (10.0%)	
typ. A.-flutt.	2 (2.0%)	1 (2.0%)	2 (2.0%)	
Lines, n (%)	11 (10.9%)	4 (7.8%)	7 (14.0%)	0.36^#^
Anterior line, n (%)	4 (4.0%)	2 (4.0%)	2 (4.1%)	0.99^#^
Cavo-tricuspid isthmus ablation, n (%)	10 (10.4%)	4 (8.3%)	6 (12.5%)	0.74^#^
Localized reentry tachycardia, n (%)	3 (3.0%)	0 (0.0%)	3 (6.0%)	0.24^#^
Sinus rhythm at the start of the study, n (%)	17 (16.8%)	9 (17.6%)	8 (16.0%)	0.99
Rhythm before cardioversion at end of procedure, n (%)				0.99^&^
Sinus rhythm	25 (24.8%)	13 (25.5%)	12 (24.0%)	
A.-fib.	75 (74.3%)	38 (74.5%)	37 (74.0%)	
Atrial tachycardia	1 (1.0%)	0 (0%)	1 (2.0%)	
Post procedural stroke n (%)	1 (1%)	1 (2%)	0 (0%)	> 0,999
AV-Fistula	1 (1%)	1 (2%)	0(0%)	> 0,999

# = Fisher’s exact test; & = Fisher-Freeman-Halton exact test.

## Complications

Procedure related complications were rare. Ischemic stroke (bilateral lower visual field defects, headache and hypertonic blood pressure with cerebral ischemia in the right media stream area, seen on MRI four days after ablation) occurred in one patient from the early ablation group (1 (2%) vs. 0(0%) *p* > 0,999). One groin complication (AV-fistula) occurred in the early ablation group (1 (2%) vs. 0(0%) *p* > 0,999). No pericardial effusion, atrio-esophageal fistula, cardiac arrest, or death occurred.

### Ablation outcomes

Ablation outcomes are shown Table 3. Freedom of any atrial arrhythmia (AT or AF) after a single ablation procedure was evaluated at 12-month follow-up based on 7-day Holter monitoring at 3-, 6-, and 12-month visits.

**Table 3:** Comparison of arrythmia recurrences rates between ablation < 12 month of > 12 after AF diagnosis.

Parameter	All patients n = 101	Ablation within 12 months n = 51	Ablation after 12 months *n* = 50	*p*-value
Recurrence after > 30 days, n (%)	39 (38.6%)	13 (25.5%)	26 (52.0%)	**0.006**
Ablation to recurrence in days, median (range) (*n* = 39 with blanking period)	176 (34–1264)	78 (37–561)	211.5 (34–1264)	0.26

The median follow-up time was 11.3 months (0.03–37.1) in patients with no documented arrhythmia recurrence. The median time to arrhythmia recurrence was 5.6 months (0.2–41.5).

**Fig. 1:**
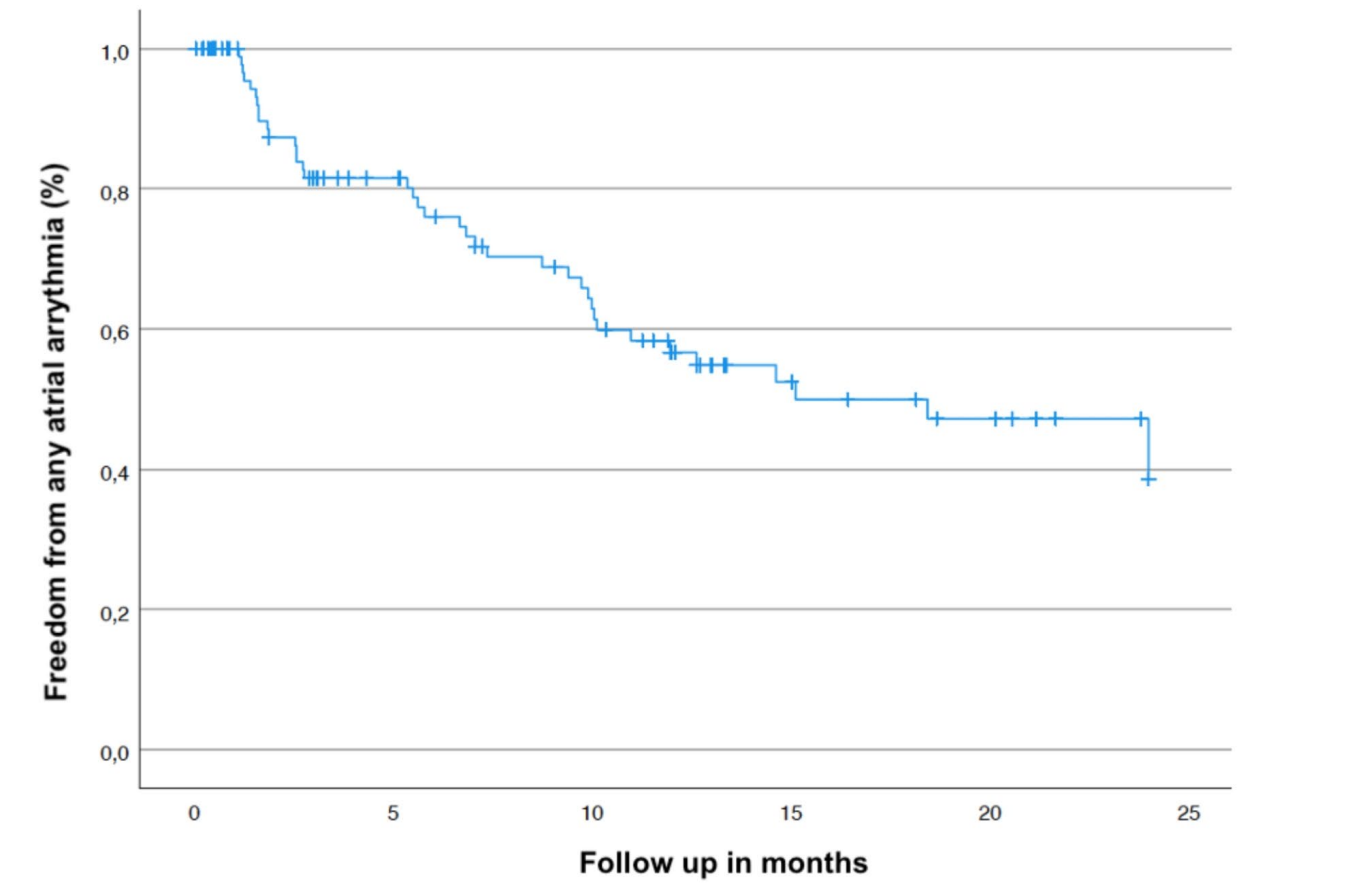
Kaplan-Mayer estimate of arrythmia recurrence of study population.

**Fig. 2:**
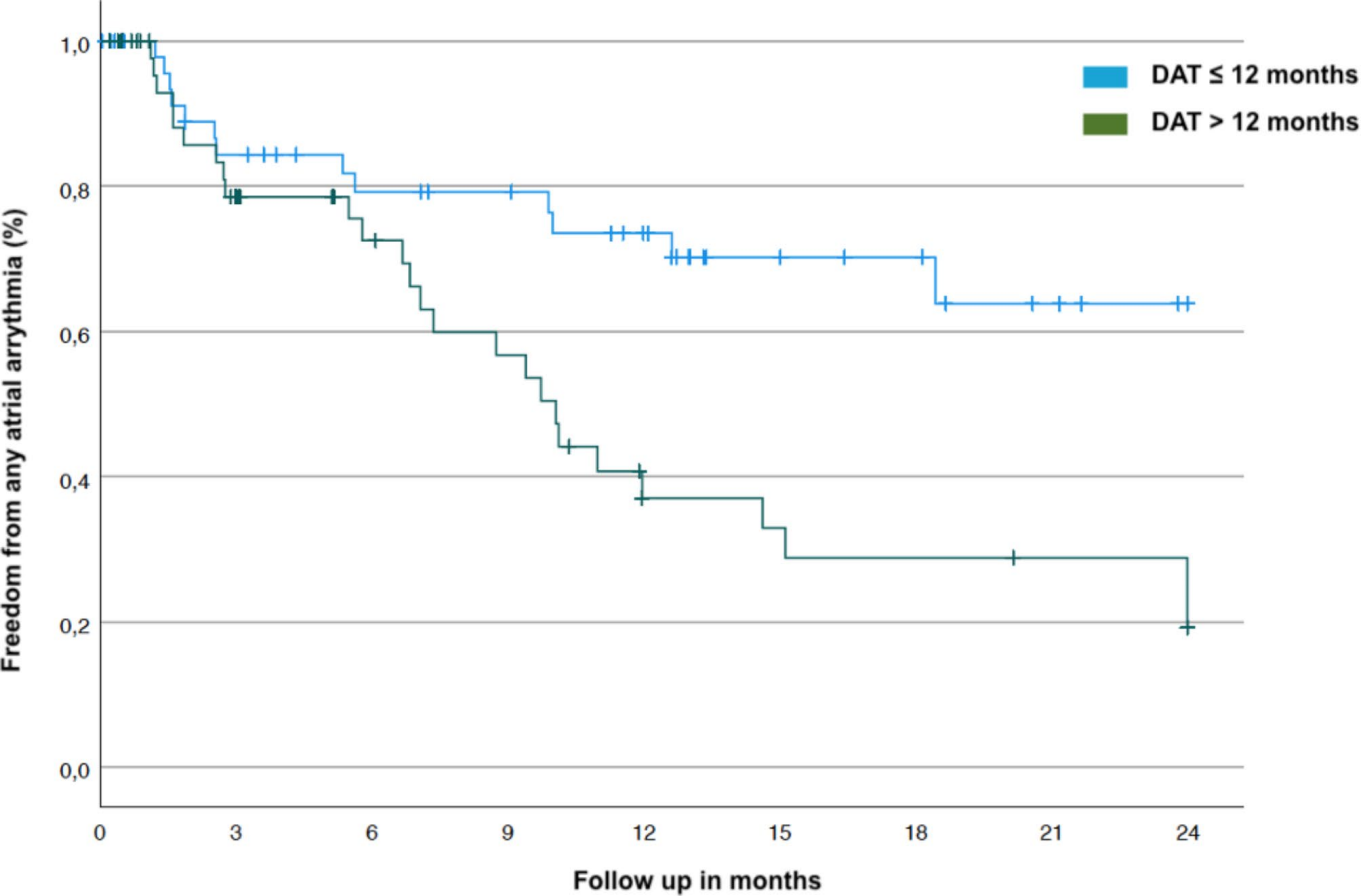
Kaplan-Mayer estimate of arrythmia recurrence divided into early ablation and late ablation group.

Recurrence of any atrial arrhythmia (AT or AF) after a 30- day blanking period occurred in 39/101 (38.6%) patients. The rate of arrhythmia recurrence after the 30-day blanking period was significantly lower in the early ablation group (≤ 12 months) as compared to the late ablation group (13/51 (25.5%) vs. 26/50 (52.0%) (log rank test; *p* = 0.003 proportional hazards assumption: *p* > 0.14). Kaplan–Meier analyses are shown in Figs. 1 and 2.

**Fig. 3:**
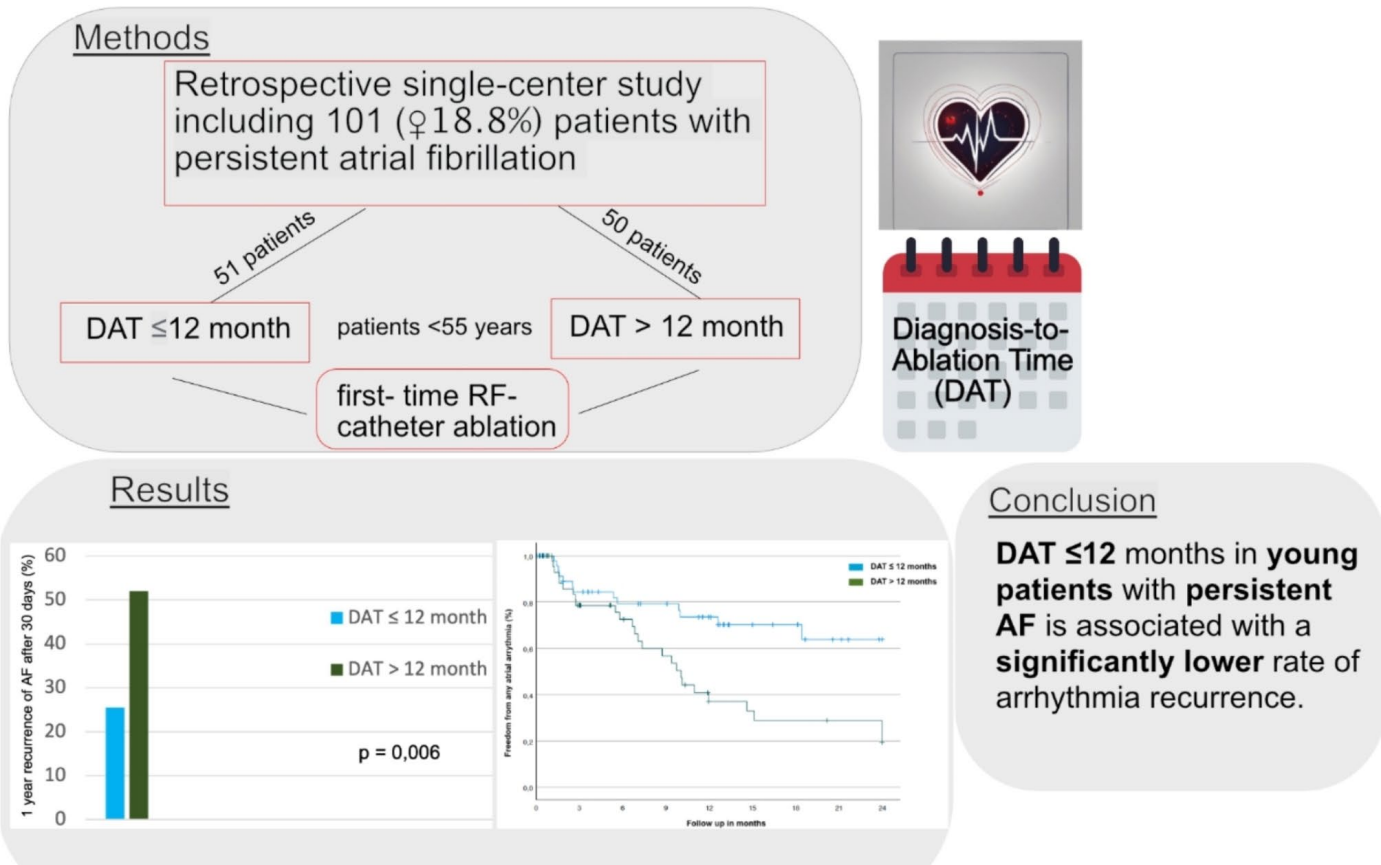
Graphical abstract.

A univariate cox regression analysis of potential predictors for arrythmia recurrence is shown in Table 4. No significant influence of BMI, left atrial size, diabetes mellitus, and congenital heart defect on atrial arrhythmia recurrences was seen. Additional left atrial substrate modification, including left atrial lines, did not result in a significantly lower rate of 1-year arrhythmia recurrence compared to PVI alone in this patient cohort (log rank test *p* = 0.2). In the multivariate analysis, catheter ablation performed > 12 months after the initial AF diagnosis was an independent predictor for the occurrence of any atrial arrythmia (OR: 2.58; (95%-CI: 1.32–5.07) (Fig. 3).

**Table 4:** Univariate regression analysis of predictors for atrial arrythmia recurrences.

	Hazard ratio (95% CI)	*p*-value
Latency from A-.fib. diagnosis to ablation in months, median (range)	1.011 (1.006–1.017) (per months of A-fib duration)	< 0.001
Female gender, n (%)	1.29 (0.589–2.86)	0.51
Age in years, median (range)	0.977 (0.932–1.023)	0.324
Body mass index in kg/m2, mean (SD)	0.967 (0.910–1.028)	0.279
Hypertension, n (%)	1.284 (0.660–2.497)	0.461
Diabetes mellitus, n (%)	0.758 (0.182–3.155)	0.704
Coronary heart disease, n (%)	0.552 (0.076–4.033)	0.558
Congenital heart defect, n (%)	0.872 (0.268–2.840)	0.82
Ejection fraction in %, median (range)	0.991 (0.951–1.034)	0.685
Left atrium, size in cm2, mean (SD)	1.010 (0.949–1.075)	0.75

## Discussion

The main finding of this study is that early AF ablation (≤ 12 months after AF diagnosis) in patients under the age of 55 years as compared to late ablation (> 12 months after AF diagnosis) leads to a significantly lower arrhythmia recurrence rate (25,5% vs. 52.0%) during long-term follow up.

Many large studies have demonstrated the efficacy and superiority of catheter ablation to AAD in patients suffering from paroxysmal and persistent AF^3,10,11^. However, as AF is more prevalent in elderly patients, reports on ablation outcomes in younger patients suffering from persistent AF are very limited. To the best of our knowledge no randomized trials exist investigating ablation outcomes in patients < 55 years.

In the present study, we investigated outcome in a younger patient population (median age of 51 years) with persistent AF, but suffering from fewer comorbidities as compared to the “usual” AF patient population. Overall freedom from any atrial arrhythmia recurrence was 61.4% which is comparable to outcomes of large randomized trials investigating a standard age AF patient population with persistent AF, where single procedure arrythmia freedom is reported between 49% and 59%^11–14^.

However, in our study early AF ablation (≤ 12 months after AF diagnosis) lead to recurrences rates of 25,5% which is low compared to larger trials with an older AF population.

In patients suffering from persistent AF, the ideal ablation strategy as well as the optimal timing of catheter ablation remains a topic of debate^11,13,15,16^. The EAST-AFNET 4 Trial which randomized AF patients (AF diagnosis ≤ 12 months) to early rhythm control or usual care demonstrated that early rhythm control (using AAD or catheter ablation) is associated with lower occurrences of adverse cardiovascular outcomes^3^. Following these findings, many studies have suggested early catheter ablation to be associated with higher rates of arrythmia freedom as well as lowering the potential progression of paroxysmal to persistent AF^15–19^. However, prospective randomized studies focusing on the optimal timing for ablation in patients suffering from persistent AF are very limited. In a large observational study from 2016 which included 1241 patients’ short diagnosis to ablation time was strongly associated with better ablation outcomes^16^.

We suspect that younger individuals might have a more resilient heart tissue and a higher capacity for recovery, making them more responsive to ablation when it is performed early in the course of the disease^20^.

Contrasting these findings, a recently published study by Kalman et al. which randomized patients with paroxysmal or persistent AF to early ablation (within one month after recruitment) or delayed ablation (at 12 months after recruitment) showed comparable results regarding arrythmia free survival between the groups^15^. However, both AF subtypes were included in the study with a higher proportion of patients suffering from persistent AF in the early ablation group. Furthermore, ablation in the delayed group was performed at 12 months after diagnosis which is still relatively early compared to the median DAT of 36 months in our study.

From the results of our study, a 12 months period may still be sufficient to provide effective outcomes of catheter ablation but that a longer DAT may significantly impair treatment success also in young and relatively healthy patients.

We speculate that factors such as structural changes of the left atrial substrate due to long term AF burden may lead to significant reduced treatment success regardless of patient age^21–23^.

### Limitations

This study was a retrospective single- center study with all implicated limitations. Persistent AF under the age of 55 years is quite rare which limits inclusion numbers. To reduce selection bias, we only included patients receiving a similar ablation approach using a HPSD energy setting. Inclusion numbers may not be adequate to draw conclusions regarding other factors influencing recurrence. Larger epidemiological studies are necessary to reliably identify possible predictors of recurrence.

It is unclear at which patients with atrial fibrillation can be classified as young, as the disease naturally occurs more frequently at elder age. However, 55 years is considered a young age for an AF patient cohort as the median age in large epidemiological studies was reported at 75 years and most recent randomized study’s included patients 60–65 years of age^12,24,25^. We chose the 55-years cut off specifically in accordance with previously published literature^26–28^.

## Conclusion

Early first-time catheter ablation in patients under the age of 55 years with persistent AF is associated with significantly lower recurrence rate of any atrial arrhythmia. Larger randomized studies are necessary to further investigate optimal timing and ablation strategy in young patients suffering from persistent AF.

## Data Availability

The datasets used and/or analysed during the current study is available from the corresponding author on reasonable request.
